# Capacity for survival in global warming: Adaptation of mesophiles to the temperature upper limit

**DOI:** 10.1371/journal.pone.0215614

**Published:** 2019-05-07

**Authors:** Tomoyuki Kosaka, Yasuyuki Nakajima, Ayana Ishii, Maiko Yamashita, Saki Yoshida, Masayuki Murata, Kunpei Kato, Yuki Shiromaru, Shun Kato, Yu Kanasaki, Hirofumi Yoshikawa, Minenosuke Matsutani, Pornthap Thanonkeo, Mamoru Yamada

**Affiliations:** 1 Life Science, Graduate School of Science and Technology for Innovation, Yamaguchi University, Yamaguchi, Japan; 2 Department of Biological Chemistry, Faculty of Agriculture, Yamaguchi University, Yamaguchi, Japan; 3 Research Center for Thermotolerant Microbial Resources, Yamaguchi, Japan; 4 Graduate School of Medicine, Yamaguchi University, Yamaguchi, Japan; 5 NODAI Genome Research Center, Tokyo University of Agriculture, Setagaya-ku, Japan; 6 Department of Bioscience, Tokyo University of Agriculture, Setagaya-ku, Japan; 7 Research Institute of Green Science and Technology, Shizuoka University, Shizuoka, Japan; 8 Department of Biotechnology, Faculty of Technology, Khon Kaen University, Khon Kaen, Thailand; Hubei University, CHINA

## Abstract

The Intergovernmental Panel on Climate Change recommends keeping the increase in temperature to less than a two-degree increase by the end of the century, but the direct impact of global warming on ecosystems including microbes has not been investigated. Here we performed thermal adaptation of two species and three strains of mesophilic microbes for improvement of the survival upper limit of temperature, and the improvement was evaluated by a newly developed method. To understand the limitation and variation of thermal adaptation, experiments with mutators and by multiple cultures were performed. The results of experiments including genome sequencing and analysis of the characteristics of mutants suggest that these microbes bear a genomic potential to endure a 2–3°C rise in temperature but possess a limited variation of strategies for thermal adaptation.

## Introduction

**A**ccording to the fifth Assessment Report of the Intergovernmental Panel on Climate Change regarding global warming over the next 100 years, in a scenario with the lowest average temperature rise, the temperature is predicted to rise by about 2 degrees, and in a scenario with the highest average temperature rise, the temperature is expected to rise by about 4 degrees. The fifth Assessment Report points out the possibility of various effects on society and the environment if the temperature continues to rise as it has been. Global warming may affect not only environments but also the diversity and populations of organisms. Each microorganism possesses a critical high temperature (CHT) that is an upper limit of survival as well as an optimum temperature for growth, probably due to the integration of various factors including general and unique metabolisms. Mesophilic microbes that have CHTs near the range of global warming would be crucially damaged by such a temperature rise. Their growth may be prevented or they may die, resulting in alterations in local ecosystems in which they are included. Alternatively, they might survive by adaptation to a temperature rise if effective mutations are accumulated. However, there has been no report on their capacity for adaptation to a rise in temperature and the variation in their strategies for adaptation, though there are many reports on *E*. *coli* regarding improvement of thermal stability and regarding the perspective of evolution to adapt to fixed or stepwise increasing temperatures [1–7]. In order to obtain clues for understanding the survival of microbes under the condition of progressive global warming and for preventing the extinction of species, we carried out *in vitro* thermal adaptation experiments using three strains of microbes with different CHTs: two strains of *Zymomonas mobilis* and one strain of *Escherichia coli*. The upper limit of thermal adaptation was further examined by using mutators that are defective in mismatch repair and thus have a high mutation frequency [8]. The strategic variation for thermal adaptation was investigated by repetitive cultivations of 4 lines of the same strain that were performed in parallel. The thermal adaptation of isolated mutants was evaluated by determination of their CHTs, by physiological characterization and by genomic sequencing analysis. The results suggested the genomic capacity of these microbes for survival in global warming. Here we also discuss functional overlapping of thermal adaptive mutations with thermotolerant genes, which are essential for survival around the CHT, in mesophilic microbes including *Z*. *mobilis* and *E*. *coli* [9–12].

## Materials and methods

### Bacterial strains and growth conditions

The microbes used in this study were *Z*. *mobilis* strains CP4, LMG 457, TISTR 548 (ATCC29191), TISTR 405, TISTR 550, TISTR 551 and NCIMB 11163, mutants of TISTR 548 and CP4, *E*. *coli* K-12 strain W3110 (IN (*rrnD-rrnE*), *rph-1*) and mutants of W3110. CP4 was obtained from H Yanase in Tottori University. LMG 457 was from the BCCM/LMG Bacteria Collection. TISTR 548, TISTR 405, TISTR 550 and TISTR 551 were from Thailand Institute of Scientific and Technological Research. NCIMB 11163 was from KM Pappas in University of Athens. W3110 was from our lab collection. *Z*. *mobilis* cells were grown in 5 ml of YPD medium (0.3% yeast extract, 0.5% peptone and 3% glucose) under a static condition, and *E*. *coli* cells were grown in 5 ml of modified Luria-Bertani (LB) medium (1% Bactotryptone, 0.5% yeast extract and 0.5% NaCl) under a shaking condition at an appropriate temperature. If necessary, streptomycin sulfate (80 μg/ml) and rifampicin (3 or 5 μg/ml) were added in agar plates.

### Thermal adaptation

**Fig 1:**
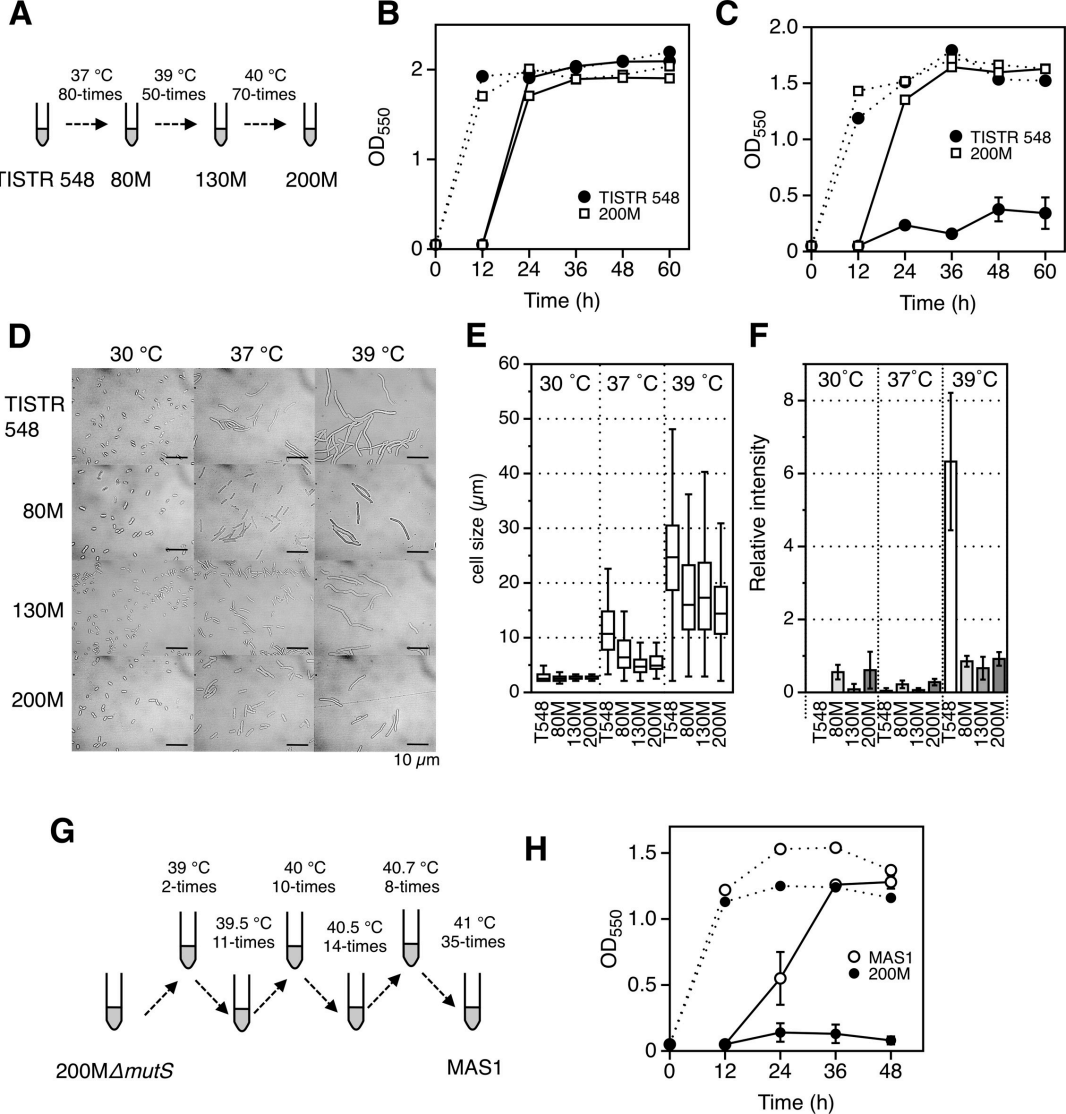
Thermal adaptation of *Z*. *moblis* TISTR548 and characteristics of thermoadapted mutants. (A) *Z*. *moblis* TISTR548 was thermally adapted as depicted, and detailed description is given in the text and the methods section. (B and C) Two-step cultivation of thermoadapted mutant 200M was performed at 30°C (B) and 40°C (C). (D to F) Cell morphology (D), cell size (E) and ROS (F) of thermoadapted mutants, 80M, 130M and 200M, were analyzed. (G and H) 200M*ΔmutS* was thermally adapted as depicted (G) and two-step cultivation of the mutant, MSA1, was performed at 41°C (H).

**Fig 2:**
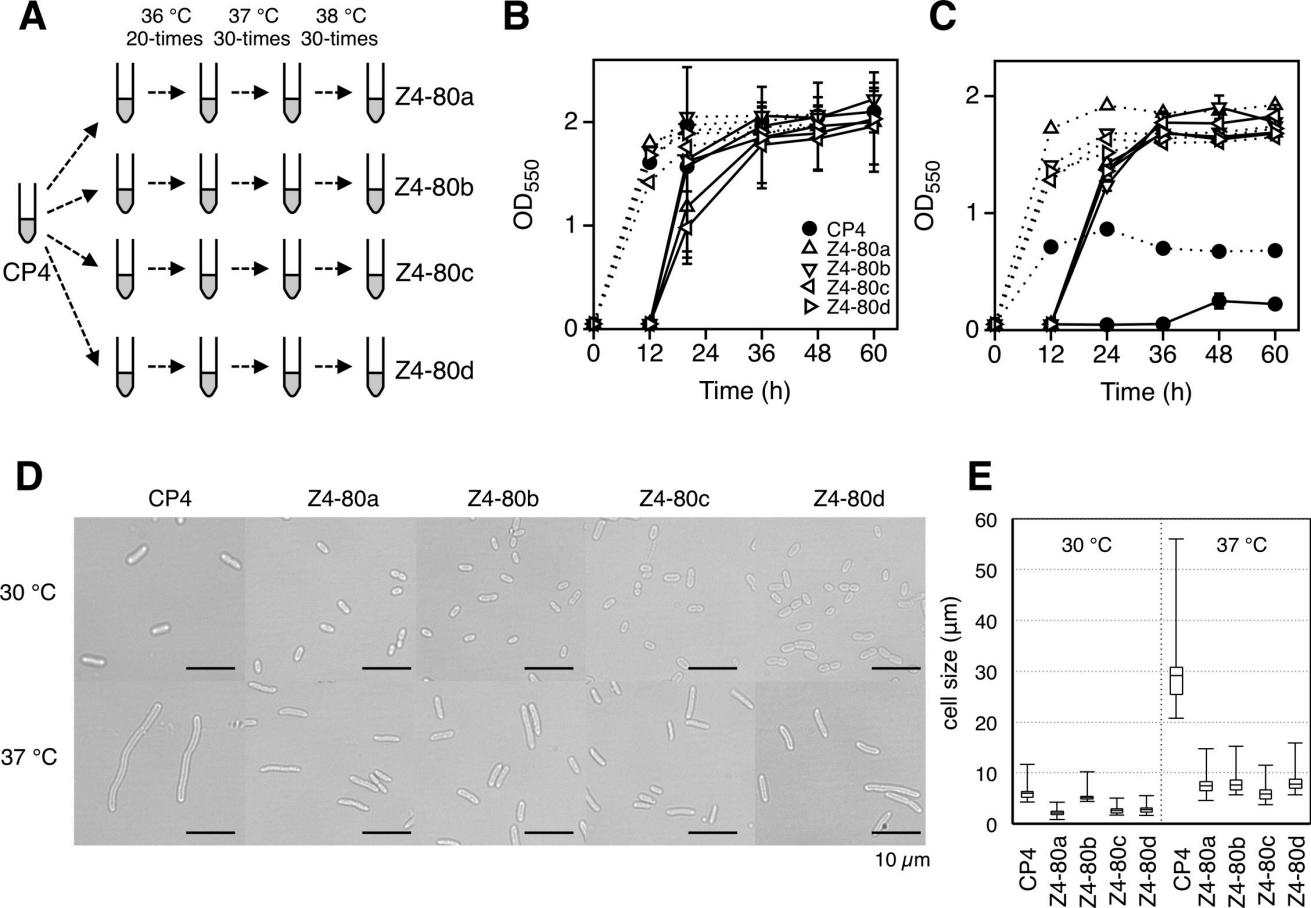
Thermal adaptation of *Z*. *moblis* CP4 and characteristics of thermoadapted mutants. (A) *Z*. *moblis* CP4 was thermally adapted in 4 lines as depicted, and detailed description is given in the text and the methods section. (B and C) Two-step cultivation of 4 thermoadapted mutants was performed at 30°C (B) and 39°C (C). (D and E) Their cell morphology (D) and cell size (E) were analyzed.

**Fig 3:**
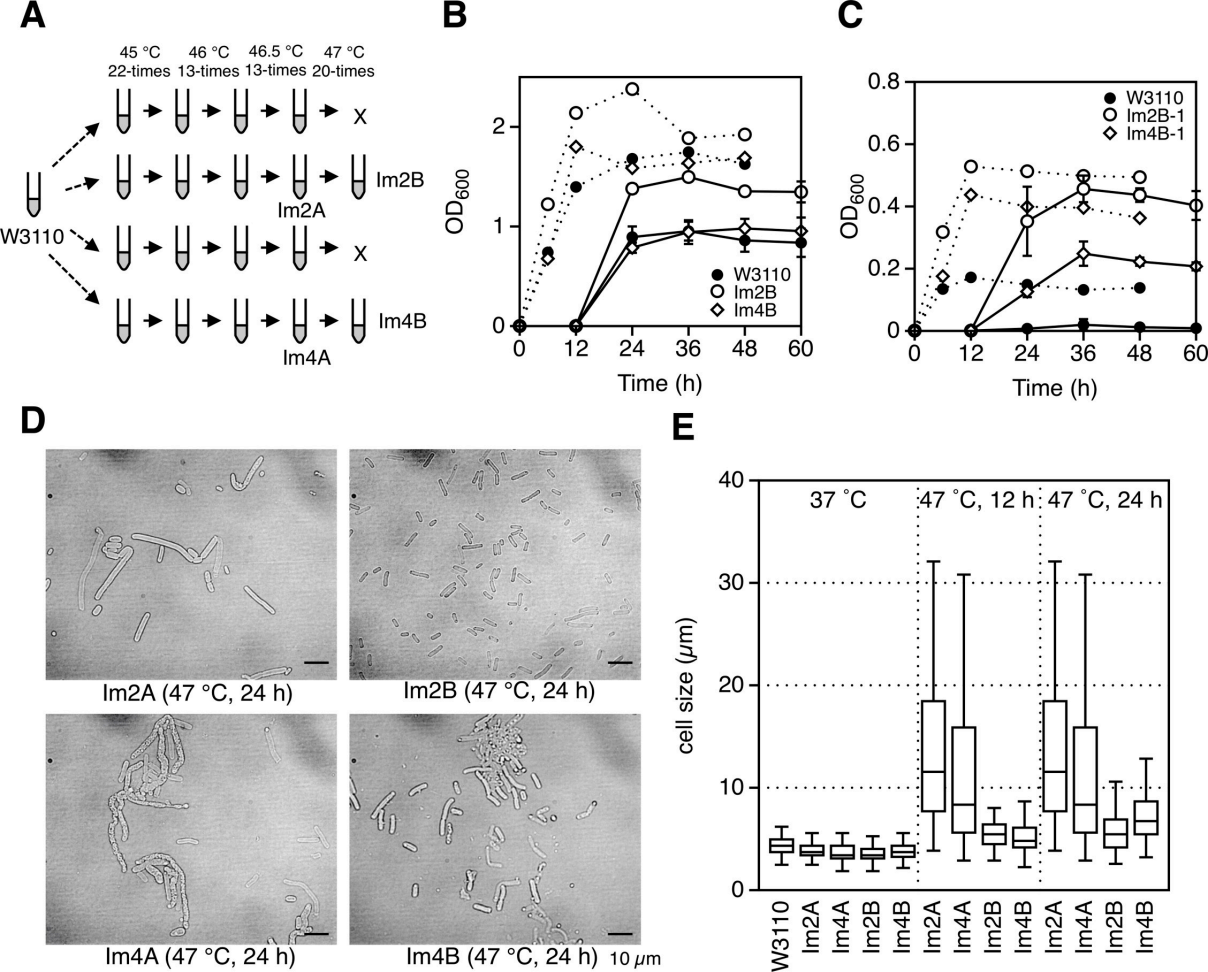
Thermal adaptation of *E*. *coli* W3110 and characteristics of thermoadapted mutants. (A) *E*. *coli* W3110 was thermally adapted in 4 lines as depicted, and detailed description is given in the text and the methods section. (B and C) Two-step cultivation was performed at 45°C (B) and 47°C (C). (D and E) Their cell morphology (D) and cell size (E) were analyzed.

Cells of *Z*. *mobilis* TISTR 548 were exposed to 80 repetitive cultivations at 37°C, 50 cultivations at 39°C and 70 cultivations at 40°C (for 12 h/each) (Fig 1A). The temperature was up-shifted when the lag time was significantly reduced. Finally, no growth was observed after 200 cultivations when the temperature was increased to 40.5°C and the repeated cultivation was therefore stopped. After single colony isolation, mutants named 80M, 130M and 200M, which were obtained from 80, 130 and 200 repetitive cultivations, respectively, were isolated. 200M was isolated after about 1,150 generations from TISTR 548. For *Z*. *mobilis* 200M-derived *mutS-* and *mutL-*disrupted mutants, 200M *ΔmutS* cells were subjected to repetitive cultivation 2 times at 39°C, 11 times at 39.5°C, 10 times at 40°C, 14 times at 40.5°C, 8 times at 40.7°C and 35 times at 41°C (for 12 h/each) (Fig 1G). On the other hand, 200M *ΔmutL* cells were subjected to repetitive cultivation 2 times at 39°C, 10 times at 39.5°C, 2 times at 40°C and 27 times at 41°C (for 12 h/each). Then MAS1 and MAL1 strains were isolated. MAL1 strain showed very slow growth at 41°C compared to that of MAS1. MAS1 was isolated after about 320 generations from 200M *ΔmutS*. For *Z*. *mobilis* CP4, thermal adaptation for 4 lines was carried out (Fig 2A). Growth change indicating thermal adaption was carefully observed. Immediately after growth improvement such as a shorter lag phase or increase in maximum turbidity had been noticed, the temperature was increased. After repeated cultivation 20 times, 30 times and 30 times at 36°C, 37°C and 38°C, respectively (80 cultivations in total), it was observed that all of the 4 independent cultures exhibited weak growth in several cultivations at 39°C. The culture at the 80^th^ cultivation, which passed about 460 generations from CP4, was then spread on YPD plates and large colonies at 39°C were isolated. For *E*. *coli* W3110, thermal adaptation for 4 lines was carried out. Cells were cultured 20 times, 13 times and 13 times at 45°C, 46°C and 46.5°C (for 12 h/each), respectively. Only 2 lines were further cultured 20 times and 15 times, respectively, at 47°C to obtain Im2B and Im4B strains (Fig 3A), which passed about 550 generations from W3110, and the remaining 2 lines were not able to grow at 47°C.

### Two-step cultivation assay

To determine the CHT of each strain, cells were subjected to two-step cultivation at the same temperature. It is assumed that cells suffer from deleterious heat stress when exposed to temperatures around their CHT. Cells would be unable to grow when the first culture performed at a temperature just above a CHT is inoculated into a fresh medium at the same temperature, but cells would be able to grow when the first culture up to a CHT is applied to the second culture (S1 Fig). In the two-step cultivation, the first cultivation was performed until a late log phase at a temperature around a putative CHT, and then a portion of the first culture was transferred into a fresh medium with OD_550_ adjusted to 0.05 and cultured at the same temperature. Cell growth was determined by measuring turbidity at OD_550_ for *Z*. *mobilis* and at OD_600_ for *E*. *coli*. Experiments were performed at least three times at the temperature for determination of the CHT for each strain. Using the culture of CP4 at 12 h in the first cultivation, the number of colony-forming units was determined as described previously [13] and a Live and Dead Cell assay was performed by the supplier’s protocol with a kit (ab115347, Abcam). These experiments were also performed at least three times.

### Construction of mutators and estimation of mutation frequency

Mutants of genes related to the mismatch repair of *Z*. *mobilis* as mutators were constructed. The 5’- and 3’-terminal regions of *mutS* and *mutL* were amplified by PCR using a set of primers (S4A Fig and S4 Table) with the genomic DNA of TISTR 548 as a template. The resultant DNA fragments were fused by the fusion PCR method using a Prime STRA HS (Takara), and the fused DNA fragment was inserted at the *attP* site of pK18-attP plasmid by BP reaction (Gateway BP Clonase II enzyme mix, Invitrogen). The pK18-attP plasmid had been constructed by insertion of the *attP* sequence into the *Hin*dIII site of pK18 [14]. The recombinant plasmid was introduced into TISTR 548 and 200M via conjugation [11] and subjected to homologous recombination, resulting in the generation of *ΔmutS*, *ΔmutL*, 200M*ΔmutS* and 200M*ΔmutL*. Their construction was confirmed by PCR using the mutant genome as a template. Apparent mutation rates of *ΔmutS* and *ΔmutL* were determined (S4B Fig). Cell cultures were appropriately diluted and spread on YPD plates or on YPD plates containing 80 μg/ml streptomycin, and CFU were then estimated after incubation at 30°C for 24 h. The apparent mutation frequency was estimated as the ratio of CFU in the presence of a drug to that in the absence of the drug. These experiments were performed at least three times.

### Growth experiments using thermoadapted mutants

For *Z*. *mobilis* strains, 10-h pre-cultured cells were inoculated into YPD medium at OD_550_ of 0.05 and cultured at an appropriate temperature under a static condition [15]. To examine the effects of acid and H_2_O_2_ stresses, pH of the growth medium was adjusted to 5.0 and 4.0 by the addition of HCl, and H_2_O_2_ was added at a final concentration of 0.5 mM, and cell growth was tested. For *E*. *coli* strains, 8-h pre-cultured cells were inoculated into LB medium at OD_600_ of 0.001 and cultured at an appropriate temperature [13]. These experiments were performed at least three times.

### Cell morphological observation and determination of cell size and ROS

Cells were collected by low-speed centrifugation, washed with phosphate buffer saline (PBS), resuspended in the same buffer, and observed under a microscope (Eclipse E600, Nikon, Japan) at × 400 magnification. Each image taken with the microscope was expanded 5 times and the cell size at the long side of more than 100 cells was measured manually. Acridine orange staining of *E*. *coli* cells was performed as follows. Cells were collected, washed with PBS, mixed with 1.0 mg/ml acridine orange solution, and incubated at room temperature for 15 min under a dark condition. Stained cells were visualized by a fluorescent microscope (Eclipse E600, Nikon, Tokyo, Japan) with Nikon B-2A (EX450-490/DM505/BA520) for fluorescein. Intracellularly accumulated ROS were detected using a fluorescence probe, 2’, 7’-dichlorofluorescin diacetate (H_2_DCFDA) [16]. Cells grown until a log phase were mixed with 0.1 mM H_2_DCFDA, incubated for 1 h, and centrifuged at a low speed to harvest cells as a pellet. The cells were then washed with PBS, suspended in the same buffer, and disrupted by sonic oscillation. The fluorescence was measured by using a POWERSCAN HT microplate reader (DS Pharma Biomedical) with excitation at 504 nm and emission at 529 nm. Protein concentration was determined by the Lowry method [17] and was used for normalization.

### Preparation of a membrane fraction and enzyme assay

A membrane fraction was prepared as described previously [15] from *Z*. *mobilis* cells that had been grown in 200 ml of YPD medium until OD_550_ of 1.5 at 30°C under a static condition. NADH oxidase activity was measured spectrophotometrically. One unit of the activity was expressed as 1 μmol of NADH oxidized per min, which was calculated with a millimolar extinction coefficient of 6.3 for NADH [15]. NADH dehydrogenase activity was measured with 2,6-Dichlorophenolindophenol (DCIP) as an electron acceptor. One unit of the activity was expressed as 1 μmol of DCIP reduced per min, which was calculated with a millimolar extinction coefficient of 14.52 for DCIP [15]. These experiments were performed at least three times.

### Preparation of genomic DNA, genomic sequencing, and genome mapping analysis

The genome DNA of *Z*. *mobislis* and of *E*. *coli* strains was extracted as described previously [18] from cells grown in YPD medium for 18 h under a static condition at 30°C and from cells grown in LB medium for 16 h under an aerobic condition at 37°C, respectively, and further purified using a Genomic-tip 20 kit (Qiagen, Hilden, Germany) according to the manufacturer’s instructions. Genome sequencing of thermoadapted mutants except for Im2B and Im2B was carried out by Illumina sequencers HiSeq and Miseq (Illumina Inc, USA) as reported previously [19]. Genome sequencing of Im2B and Im2B was performed by an Ion Torrent PGM sequencer (Thermo Fisher Scientific Inc) according to the manufacturer's instructions in the DNA Core Facility of the Center for Gene Research, Yamaguchi University. Accession numbers of all sequence data and platforms are shown in S5 Table.

For genome mapping analysis, three reference genome sequences, *Escherichia coli* str. K-12 substr. W3110 (GenBank acc. No: AP009048.1), *Zymomonas mobilis* subsp. mobilis ATCC 29191 (GenBank acc. No: CP003704.1-CP003707.1), and *Zymomonas mobilis* subsp. mobilis str. CP4 (GenBank acc. No: CP006818.1 and CP006891.1-CP006895.1), were downloaded from NCBI ftp site, ftp://ftp.ncbi.nlm.nih.gov/. The Illumina and Ion torrent PGM sequencing reads were aligned with each of the reference genome sequences by using Burrows-Wheeler Aligner (BWA, version 0.6.2) and mappers of Torrent Suite 3.4.1 (TMAP-TS3.4.1), respectively [20]. The mutation sites were searched for by using the method reported previously [21]. All mutations in coding regions in all adapted mutants were confirmed by the Sanger method [22] after amplification of individual regions by PCR using primers listed in S6–S8 Tables.

## Results

### Determination of CHT

Different strains in the same species exhibit different sensitivities to a high temperature. To determine such differences, a simple method, called two-step cultivation, for determination of CHT was developed (S1 Fig). When examined by this method at different temperatures, 30–39°C, *Z*. *mobilis* CP4 showed no growth in the second culture at 38°C and 39°C but showed growth at 37°C or a lower temperature (Fig A in S2 Fig). Colony-forming ability and live and dead cell analyses revealed that most of the cells in the first culture above their CHT died (Figs B and C in S2 Fig). Notably, cells grown at 37°C, a temperature close to their CHT, were stained red as well as green, presumably due to some membrane damage at that temperature that allowed a red fluorescent dye to enter the cytoplasm, but the cells showed colony-forming units (CFU) about 3 orders of magnitude larger than those grown at 38°C. Similarly, the CHT of LMG 457 was determined to be 37°C and the CHTs of TISTR 548, TISTR 405, TISTR 550, TISTR 551 and NCIMB 11163 were all 38°C.

### Thermal adaptation of *Z*. *mobilis* TISTR 548 and mutant characteristics

One of issues in this study was whether microbes have a limitation to their adaptation to a high temperature or whether mesophiles are able to evolve to become thermo-resistant at the thermophile level by accumulation of mutations. We thus attempted to clarify the potential of microbes for thermal adaptation. *Z*. *mobilis* TISTR 548 was exposed to repetitive cultivation with a gradual increase in temperature (Fig 1A), and thermoadapted mutants, called 80M, 130M and 200M, were obtained. Two-step cultivation analysis revealed that the CHTs of all three mutants were 40°C (Table 1 and Fig 1B and 1C), though 130M and 200M exhibited faster growth and higher turbidity at 40°C than those of 80M. Since 130M and 200M exhibited almost the same levels of turbidity in the second cultivation, it is likely that thermal adaptation by repetitive cultivation had reached the limit. In addition to enhanced thermotolerance, 130M and 200M became resistant to a high concentration of glucose but sensitive to a low pH compared to the resistance and sensitivity of the parental strain (Fig A in S3 Fig). The cell morphology of *Z*. *mobilis* was changed at a temperature close to the CHT and the cells became filamentous. The cell lengths of the thermoadapted mutants 80M, 130M and 200M, however, were significantly smaller than the cell length of the parental strain at 37°C and 39°C, whereas the cell lengths of all strains were almost the same at 30°C (Fig 1D and 1E). Therefore, it is likely that cell elongation at a high temperature is suppressed by thermal adaptation. Cells are exposed to oxidative stress at a temperature close to their CHT [23, 24]. Consistently, TISTR 548 showed remarkable accumulation of reactive oxygen species (ROS) at 39°C, but all of the thermoadapted mutants showed a low level of ROS accumulation (Fig 1F). We measured respiratory activity because it has been reported that reduction of NADH dehydrogenase activity in the respiratory chain is somehow linked to enhancement of thermotolerance [25]. All of the thermoadapted mutants exhibited only about half of the NADH dehydrogenase and oxidase activities of the parental strain (Fig B in S3 Fig). Therefore, it is likely that one of the reasons for the thermal adaptation in these mutants is a low level of NADH dehydrogenase activity, which may reduce leakage of electrons and thus prevent the accumulation of ROS. Genomic sequencing analysis revealed that 200M has 17 mutated genes including missense mutations and transposon insertions (S1 Table and Table 1).

**Table 1:** CHTs and mutations of thermoadapted mutants.

Strain	CHT	Number of mutation (coding region)	Chromosome^a^			
			Transition	Transversion	InDel	Tn
*Z*. *mobilis* TISTR 548	38°C					
*Z*. *mobilis* TISTR 548 200M	40°C	21 (12)	6	4	5	6
*Z*. *mobilis* TISTR 548 200MMAS1	41°C	24 (15)	9	4	5	6
*Z*. *mobilis* CP4	37°C					
*Z*. *mobilis* CP4 Z4-80a	39°C	5 (5)	5	0	0	0
*Z*. *mobilis* CP4 Z4-80b	39°C	4 (2)	1	0	3	0
*Z*. *mobilis* CP4 Z4-80c	39°C	8 (4)	4	0	4	0
*Z*. *mobilis* CP4 Z4-80d	39°C	4 (3)	3	0	1	0
*E*. *coli* W3110	45°C					
*E*. *coli* W3110 Im2B	47°C	6 (4)	2	2	2	0
*E*. *coli* W3110 Im4B	47°C	9 (6)	4	2	3	0

^a^ Numbers were from S1 Table to S3 Table.

### Further thermal adaptation with *ΔmutS* and *ΔmutL* as mutators

The thermal adaptation in this study depended on accumulation of mutations during repetitive cultivation, and increased mutation frequency may thus allow for the creation of further thermotoletant mutants, which in turn would lead to an understanding of the potential of the intrinsic genome for thermal adaptation. Disrupted mutations of genes for mismatch repair, *ΔmutS* and *ΔmutL*, which increased the level of mutation frequency by more than 2 orders of magnitude (S4 Fig), were thus introduced into 200M. Two lines of both 200M*ΔmutS* and 200M*ΔmutL* as mutators were subjected to thermal adaptation by repeated cultivation with a gradual increase in temperature from 39°C to 41°C (Fig 1G). After cultivation for 80 times, one adapted mutant, MAS1, from the two lines of 200M*ΔMutS* was obtained, but no adapted mutant was obtained from the lines of 200M*ΔMutL*. MAS1 was found to have a CHT at 41°C, which is a one-degree improvement from that of 200M (Fig 1H and Table 1), and to have acquired 3 missense mutations (S1 Table and Table 1). Eventually, the thermoresistance of *Z*. *mobilis* TISTR 548 could not be improved by more than three degrees even with application of mutators.

### Thermal adaptation of *Z*. *mobilis* CP4 and mutant characteristics

Thermal adaptation was also attempted with *Z*. *mobilis* CP4, for which the CHT is one degree lower than that of TISTR 548. In this case, to determine whether there are common mechanisms for thermal adaptation as the second issue, repetitive cultivations of 4 lines were performed in parallel (Fig 2A). Eventually, 4 thermoadapted strains designated as Z4-80a, Z4-80b, Z4-80c and Z4-80d were obtained from 4 independent cultures and they were found to have CHTs at 39°C (Fig 2B and 2C and Table 1). The cell shape of the parental strain became filamentous at 37°C, but all of the thermoadapted strains showed almost the same cell sizes as those at 30°C (Fig 2D and 2E), as did thermoadapted mutants from TISTR 548, and reduced accumulation of ROS at 37°C (Fig A in S5 Fig). Interestingly, two of the 4 mutants and the other two mutants exhibited similar phenotypes. Z4-80a and Z4-80c showed greater resistance to H_2_O_2_ (Fig B in S5 Fig) and to rifampicin (Fig C in S5 Fig), whereas Z4-80b and Z4-80d were more resistant to high concentrations of glucose and streptomycin (Fig C in S5 Fig) and showed lower activities of NADH dehydrogenase and NADH oxidase (Fig C in S5 Fig). The latter respiratory characteristics are similar to those of 200 M but not to those to Z4-80a and Z4-80c. Consistently, genomic analysis revealed that Z4-80a and Z4-80c shared 4 mutated genes, though the mutation points were different (one gene being the same), and that Z4-80a bore one additional mutated gene (S2 Table). On the other hand, Z4-80b and Z4-80d had no shared mutated genes, but the possibility that the combination of mutations allows the two mutants to exhibit similar phenotypes cannot be excluded.

### Thermal adaptation of *E*. *coli* W3110 and mutant characteristics

*E*. *coli* is relatively heat-resistant among mesophilic microbes and its CHT is 7–8°C higher than that of *Z*. *moblis*. A thermal adaptation experiment using *E*. *coli* was thus thought to be important to understand the impact of global warming on mesophiles. In contrast to the static cultivation used for *Z*. *moblis* strains, *E*. *coli* W3110 was repeatedly cultivated under a shaking condition at temperatures from 45°C to 47°C. Although the experiment was initiated by 4 independent cultures, mutants of Im2A and Im4A were obtained from the remaining two lines (Fig 3A). The CHT of both thermoadapted mutants was 2°C higher than that of the parental strain (Fig 3B and 3C and Table 1). The cells of both thermoadapted mutants were obviously smaller than those of intermediate mutants, Im2A and Im4A, when exposed to a high temperature of 47°C (Fig 3D and 3E), at which the parental strain was unable to grow. Genomic sequence analysis revealed that Im2B and Im4B had 4 and 6 missense mutations, respectively (S3 Table). Staining of cells grown at 37°C with acridine orange showed that staining of the cells of both mutants was weaker than that of the parental strain (Fig A in S6 Fig), indicating that their amounts of RNA and/or DNA were small and that their turbidity of culture was lower when grown at 37°C (Fig B in S6 Fig). These findings might be due to mutations of *spoT* that were only shared in both mutants because some *spoT* mutants have been shown to reduce RNA and DNA syntheses via accumulation of ppGpp [26]. Taken together, the results indicate that Im2B and Im4B have similar phenotypes. Notably, two stepwise thermoadapted *E*. *coli* mutants reported independently [2, 4] share three mutated genes including *spoT* in more than 26 mutated genes, suggesting that it is a crucial target for thermal adaptation in *E*. *coli*. However, comparable phenotypes of these mutants are not available.

### Mutation spectra of thermoadapted mutants

The mutations that accumulated via thermal adaptation in the three tested strains include a single nucleotide substitution (SNS) and an indel and transposon (Tn) insertion, but their mutation spectra are different (Table 1). There is no Tn insertion mutation in thermoadapted mutants from *Z*. *mobilis* CP4 and *E*. *coli* W3110, though SNSs and indel(s) are present in those from all three strains. Ratios of mutation number per generation are 1.9 x 10^−2^ in 200M, 1.1–1.7 x 10^−2^ in CP4-derived mutants and 1.1–1.6 x 10^−2^ in W3110-derived mutants. The rates in *Z*. *mobilis* tend to be higher than those in *E*. *coli*, and the latter is higher than those in previous reports on *E*. *coli*: 0.15–0.35 x 10^−2^ for experiments with 2,000 generations at 42°C [7] and 0.67 x 10^−2^ for experiments with 5,000 generations at a stepwise temperature upshift from 36.9°C to 44.8°C (before a mutator occurred) [2]. At least in *E*. *coli*, mutation frequency might thus be higher around the CHT. Although all of the adapted mutants in this study have almost preferentially transition mutations as SNS, G/C to A/T and A/T to G/C are dominant in *Z*. *mobilis* thermoadapted mutants and *E*. *coli* thermoadapted mutants, respectively. Synonymous mutations are rare in thermoadapted mutants: none of the 13 SNSs in protein-coding regions in CP4-derived mutants (in total) and none of the 7 SNSs in protein-coding regions in W3110-derived mutants. Thus, the ratios of synonymous to nonsynonymous mutations are significantly less than the ratio of 24.9% expected by chance that is adapted from ref. 7. Thus, these data suggest a strong signal of adaptive evolution. The thermoadapted mutants obtained in this study bear 2–15 mutations in protein-coding regions of genes, which are categorized into 6 groups (Table 2). Most of them are overlapping with the common classification for thermotolerant genes required for survival at the CHT in *Z*. *mobilis* TISTR 548, *E*. *coli* W3110 and *Acetobacter tropicalis* SKU1100 [9–12]. This overlapping may suggest that mutations generated by thermal adaptation enhance physiological functions of products of thermotolerant genes or their closely related genes, particularly enhancement of membrane stabilization or maintenance of membrane potential or intracellular solute amount or composition.

**Table 2:** Classification of mutated genes in thermoadapted mutants.

Classification	*Z*. *mobilis* TISTR 548^a^		*Z*. *mobilis* CP4^a^				*E*. *coli* W3110^a^	
	200M	MAS1	Z4-80a	Z4-80b	Z4-80c	Z4-80d	Im2B	Im4B
General metabolism	1	2	0	1	0	1	1	1
Membrane stabilization	3	3	1	0	1	1	0	2
Protein quality control	1	2	0	0	0	0	0	0
Transcriptional regulation	4	5	1	1	1	0	2	2
Transporter	2	2	3	0	2	1	0	1
Others	1	1	0	0	0	0	0	0

^a^ Numbers were from S1 Table to S3 Table.

It is assumed that not all of these mutations contribute to improvement of thermoresistance and that some of them were acquired independently of thermal adaptation. Frame-shift mutations by indels and Tn insertion in protein-coding regions would mostly generate negative or neutral effects on their thermoresistance. No large deletion was detected in this study, possible contributing to thermal adaptation as demonstrated in other bacteria [27]. Preliminary experiments, in which individual mutations of CP4-derived thermoadapted mutants were introduced into the genome of the parental CP4 by the procedure similar to that for construction of mutators (see S4 Fig) after cloning each mutated gene into the pK18-attP plasmid (followed by confirmation by nucleotide sequencing), showed that most of single mutations in coding regions of genes for categories of general metabolism, membrane stabilization and transcriptional regulation (Table 2) improved the CHT by about 1 degree, which was determined by the two-step cultivation method in comparison with the corresponding thermoadapted mutant and CP4. These genes were ZCP4_1739 and ZCP4_0567 among 5 mutants tested for Z4-80a, ZCP4_0028 and ZCP4_1646 among 2 mutants tested for Z4-80b, ZCP4_1739 and ZCP4_0567 among 4 mutants tested for Z4-80c, and ZCP4_1739 and ZCP4_0588 among 3 mutants tested for Z4-80d (S2 Table). Therefore, it is assumed that a few mutations among many mutations are valuable and beneficial for high-temperature tolerance characteristics and that effective mutations of these mutants are mostly gain-of-function mutations.

## Discussion

Previous adaptation experiments were carried out by repetitive cultivations in minimum or rich media at fixed temperatures from 20°C to 42°C [3, 5–7] or by a stepwise increase in temperature to 45.5°C [2] or 48.5°C [1, 4], and thermoadapted mutants isolated by a stepwise temperature upshift in a rich medium were shown to survive at equivalent high temperatures in a minimum medium [1]. In this study using thermal adaptation experiments in a rich medium with two different species and three strains with distinct CHTs, there was an improvement in the CHTs up to 2 degrees (Table 1) and application of mutators to further improve the CHT resulted in an increase of one more degree in the CHT of *Z*. *mobilis* TISTR 548, thus giving a total increase of 3 degrees in the CHT. The increase in CHT of 3 degrees may be close to the limit of thermal adaptation considering the application of a mutator. Therefore, it is assumed that the CHT improvement potential in the genome is around 3 degrees in these mesophiles. Although there is no information on generation speed or mutation rate in nature, an increase of 2–3 degrees in global temperature is considered to have a strong impact on mesophiles and the ecosystems including these microbes.

In *in vitro* experiments, cell density in a rich medium becomes 10-100-times higher than that in a minimum medium, while the mutation rates under phosphate- limited and carbon-limited conditions are 9-times and 6-times higher, respectively, than the mutation rate under a nutrient-sufficient condition [28]. In nature, microbes may rarely be in a nutrient-rich environment and are generally exposed to conditions in which several nutrients are limited. Therefore, the mutation frequency in nature would be within the range of mutation rates in the background of a mismatch-repair defect (mismatch repair mutant), but acquisition of thermal adaptation is mainly dependent on chances to obtain nutrients for proliferation. On the other hand, thermal adaptation with multiple lines of the same parental strain provides an intriguing conjecture that there is a low diversity of thermal adaptation strategies. Two of the *Z*. *mobilis* thermoadapted mutants accumulated mutations in almost the same genes and the remaining two mutants exhibited similar phenotypes, though they had mutations in different genes. Two *E*. *coli* thermoadapted mutants derived from 4 lines shared phenotypes and had mutations in the same or similar function-related genes. These findings lead us to speculate that the limited diversity for thermal adaptation decreases the chance for microbes to survival in the progress of global warming.

There are some key issues to be clarified. The period of adaptation in this study could be considered to be too short for acquisition of beneficial mutations. Thermoadaptation of more than 160 generations (about 400 generations in the case of TISTR548) was continued after reaching the upper limit temperature, but no more improvement of thermoresistance was found. However, we cannot exclude the possibility of accumulation of beneficial mutations in a much longer period of adaptation than that tested in this study. The thermal adaptation experiments performed in this study are very simple compared with complicated nature, in which there are seasonal temperature oscillations and spatial variation with regular cooler periods/places that are still suitable for the growth of mesophiles. Thus, if mesophiles in an area are continuously exposed to high temperatures in a range exceeding their CHTs for a duration that is sufficient to kill them, they will be removed from that area.

## Supporting information

S1 Fig Two-step cultivation for determination of CHT. (A and B) Strains A and B are cultured in a rich medium at 37°C (A) and 38°C (B) as the first cultivation (straight lines). After 12 h, an aliquot of the culture is transferred to a fresh medium and subjected to the second cultivation (dotted lines). Strain B shows an increase in turbidity (OD_550_) at 37°C but not at 38°C, indicating that its CHT is 37°C.
(PDF)

S2 Fig Determination of CHT of *Z*. *moblis* CP4. (A) CHT of *Z*. *moblis* CP4 was determined by the two-step cultivation method as described in S1 Fig. (B and C) Using the culture of CP4 at 12 h in the first cultivation, a live and dead cell assay and determination of CFU were performed.
(PDF)

S3 Fig Characteristics of thermoadapted mutants from TISTR548. (A) Effects of a high concentration of glucose and low pHs on growth of thermoadapted mutants from TISTR548. Mutants, 80M, 130M and 200M, and their parental strain were grown at 30°C in YPD medium until the exponential phase. The culture was serially diluted and spotted on pH 7-adjusted YP plates containing 3% glucose, on pH 7-adjusted YP plates containing 12% glucose, on pH 5-adjusted YP plates containing 3% glucose and on pH 4-adjusted YP plates containing 3% glucose, which were then incubated for 48 h. (B) Respiratory activities of thermoadapted mutants from TISTR548. Thermoadapted mutants, 80M, 130M and 200M, and their parental strain were grown at 30°C in YPD medium under a static condition, and their membrane fractions were prepared. NADH dehydrogenase and NADH oxidase activities in the membrane fractions were then determined.
(PDF)

S4 Fig Procedure for construction of mutators, 200M*ΔmutS* and 200M*ΔmutL*, and their mutation frequency. (A) An upstream DNA fragment including the coding sequence for the N-terminal portion of MutS and a downstream DNA fragment including the coding sequence for the C-terminal portion of MutS were separately amplified by PCR with the genomic DNA of TISTR 548 as a template and appropriate primers, and the two fragments were connected together by fusion PCR. The resultant *ΔmutS-attB* PCR fragment was inserted into pK18-attP, which was used for construction of *ΔmutS* and 200M*ΔmutS* as described in the methods section. Similarly, *ΔmutL* and 200M*ΔmutL* were constructed. (B) Mutation rate was determined as the ratio of apparent mutation frequency of *ΔmutS* or *ΔmutL* to that of their parent.
(PDF)

S5 Fig Characteristics of thermoadapted mutants from CP4. (A) Comparison in the accumulation of ROS. Thermoadapted mutants, Z4-80a, Z4-80b, Z4-80c and Z4-80d, from CP4 were grown at 30°C, 36°C and 38°C in YPD medium. Using cells at the exponential phase, fluorescent intensity with H_2_DCFDA, which reflects accumulation of ROS, was measured. (B) Resistance to H_2_O_2_ among thermoadapted mutants from CP4. Cells that were similarly grown at 30°C in TPD medium until the exponential phase were inoculated and grown at 30°C, 36°C and 38°C in YPD medium with or without 0.5 mM H_2_O_2_ for 24 h. The turbidity at 24 h was measured. (C) Effects of a high concentration of glucose and antibiotics on growth of thermoadapted mutants. Cells were grown at 30°C in YPD medium until the exponential phase. The culture was serially diluted and spotted on YPD plates containing 12% glucose or antibiotics and incubated at 30°C for 24 h. (D) Respiratory activities of thermoadapted mutants. Cells were grown at 30°C in YPD medium under a static condition, and their membrane fractions were prepared. NADH dehydrogenase (DCIP reductase) and NADH oxidase activities in the membrane fractions were then determined.
(PDF)

S6 Fig Comparison of acridine orange-stained cells and growth of thermoadapted mutants from W3110 with those of the parent. (A) Thermoadapted mutants, ImB2 and ImB4, from W3110 were grown at 47°C in LB medium and cells at the exponential phase were stained with acridine orange. (B) Cell growth was compared by cultivation at 37°C in LB medium.
(PDF)

S1 Table Summary of mutations in thermoadapted mutants from *Zymomonas mobilis* TISTR548. (PDF)

S2 Table Summary of mutations in thermoadapted mutants from *Zymomonas mobilis* CP4. (PDF)

S3 Table Summary of mutations in thermoadapted mutants from *Escherichia coli* W3110. (PDF)

S4 Table Primers used for construction of mutators. (PDF)

S5 Table List of sequencing data and platform used in this study. (PDF)

S6 Table Primers used for confirmation of mutation sites in coding regions of thermoadapted mutants from *Z*. *mobilis* TISTR548. (PDF)

S7 Table Primers used for confirmation of mutation sites in coding regions of thermoadapted mutants from *Z*. *mobilis* CP4. (PDF)

S8 Table Primers used for confirmation of mutation sites in coding regions of thermoadapted mutants from *E*. *coli* W3110. (PDF)
