# Correction: Capacity for survival in global warming: Adaptation of mesophiles to the temperature upper limit

**DOI:** 10.1371/journal.pone.0218985

**Published:** 2019-06-20

**Authors:** Tomoyuki Kosaka, Yasuyuki Nakajima, Ayana Ishii, Maiko Yamashita, Saki Yoshida, Masayuki Murata, Kunpei Kato, Yuki Shiromaru, Shun Kato, Yu Kanesaki, Hirofumi Yoshikawa, Minenosuke Matsutani, Pornthap Thanonkeo, Mamoru Yamada

The tenth author's name is spelled incorrectly. The correct name is: Yu Kanesaki.
