# Supplementary figures and images for: Capacity for survival in global warming: Adaptation of mesophiles to the temperature upper limit
Source: PLoS One. 2019 May 7;14(5):e0215614. doi: 10.1371/journal.pone.0215614 (PMC6504187; doi:10.1371/journal.pone.0215614)

A

37 °C

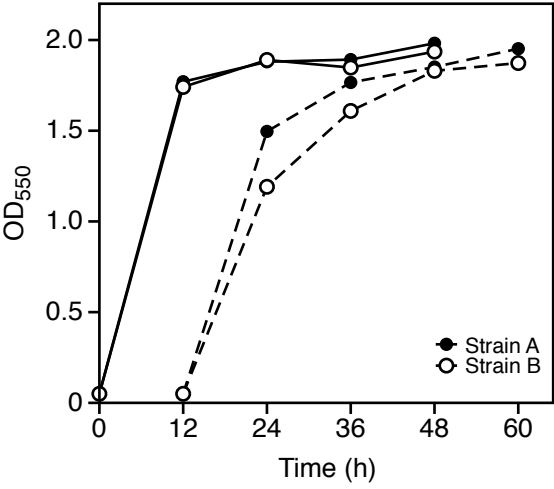

B

38 °C

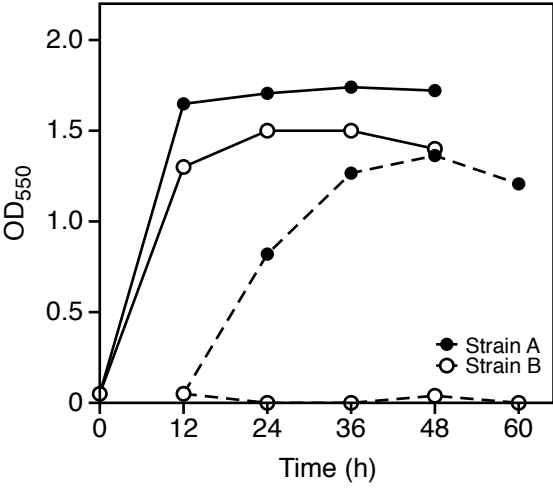

Supplement: S1 Fig — (A and B) Strains A and B are cultured in a rich medium at 37°C (A) and 38°C (B) as the first cultivation (straight lines). After 12 h, an aliquot of the culture is transferred to a fresh medium and subjected to the second cultivation (dotted lines). Strain B shows an increase in turbidity (OD550) at 37°C but not at 38°C, indicating that its CHT is 37°C. (PDF) [file pone.0215614.s001.pdf]

A

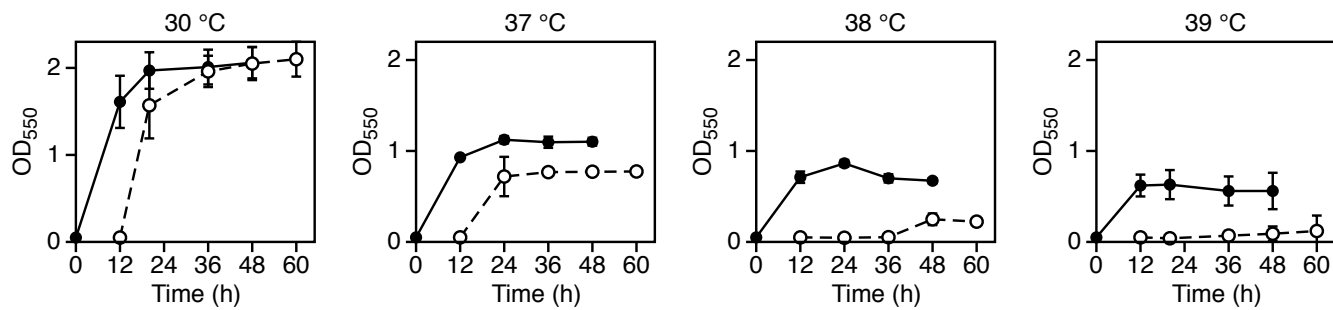

B

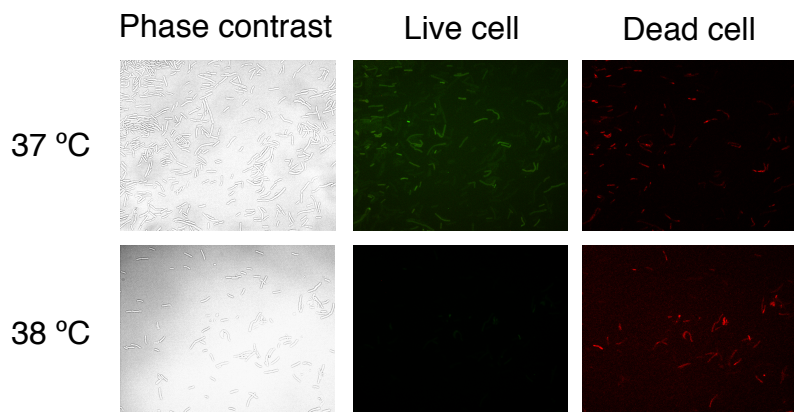

C

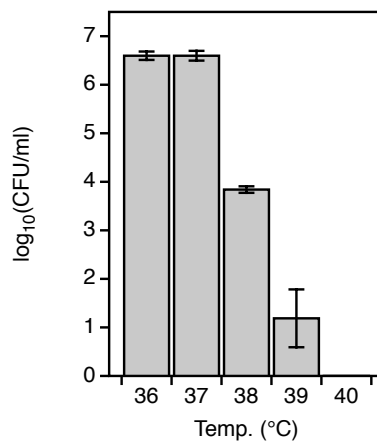

Supplement: S2 Fig — (A) CHT of Z. moblis CP4 was determined by the two-step cultivation method as described in S1 Fig. (B and C) Using the culture of CP4 at 12 h in the first cultivation, a live and dead cell assay and determination of CFU were performed. (PDF) [file pone.0215614.s002.pdf]

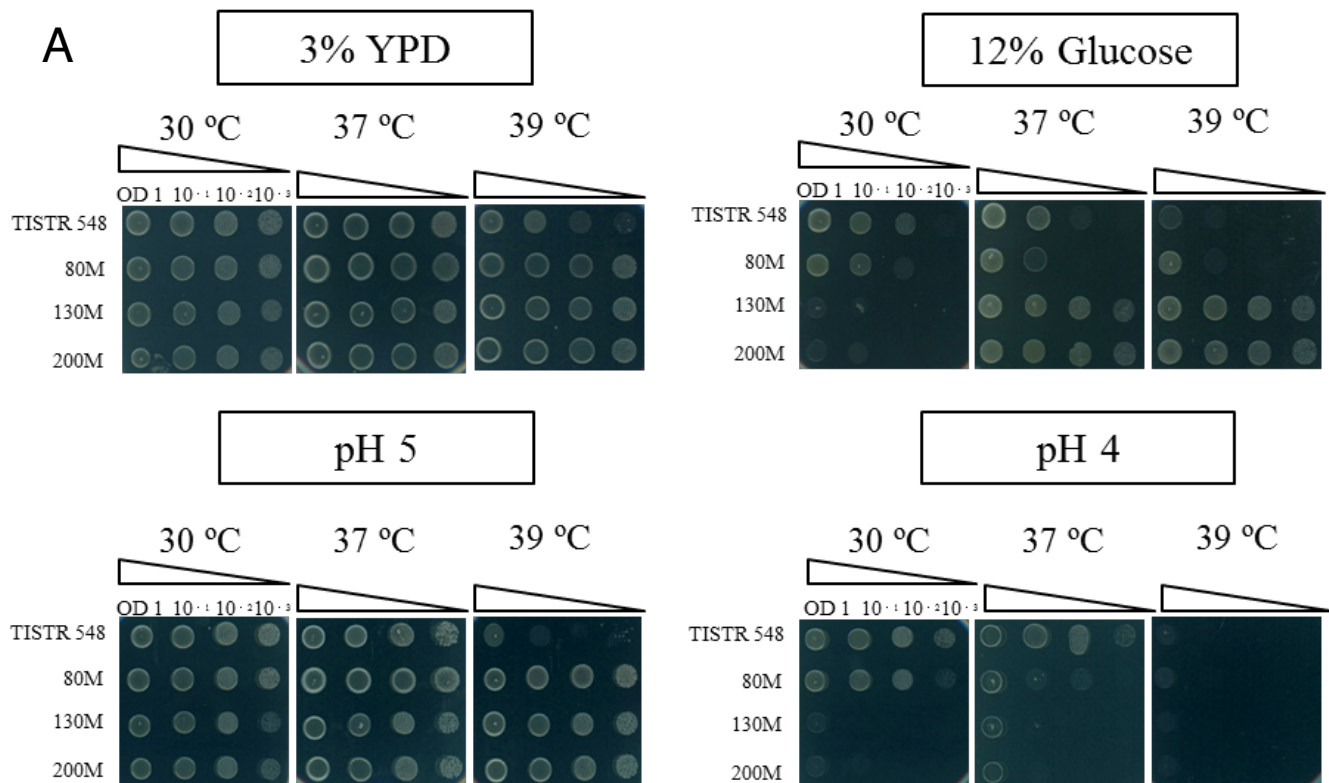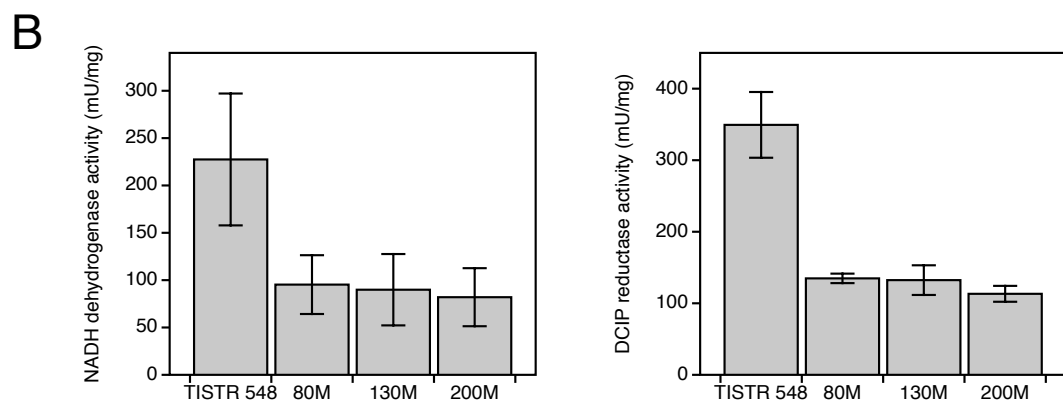

Supplement: S3 Fig — (A) Effects of a high concentration of glucose and low pHs on growth of thermoadapted mutants from TISTR548. Mutants, 80M, 130M and 200M, and their parental strain were grown at 30°C in YPD medium until the exponential phase. The culture was serially diluted and spotted on pH 7-adjusted YP plates containing 3% glucose, on pH 7-adjusted YP plates containing 12% glucose, on pH 5-adjusted YP plates containing 3% glucose and on pH 4-adjusted YP plates containing 3% glucose, which were then incubated for 48 h. (B) Respiratory activities of thermoadapted mutants from TISTR548. Thermoadapted mutants, 80M, 130M and 200M, and their parental strain were grown at 30°C in YPD medium under a static condition, and their membrane fractions were prepared. NADH dehydrogenase and NADH oxidase activities in the membrane fractions were then determined. (PDF) [file pone.0215614.s003.pdf]

A

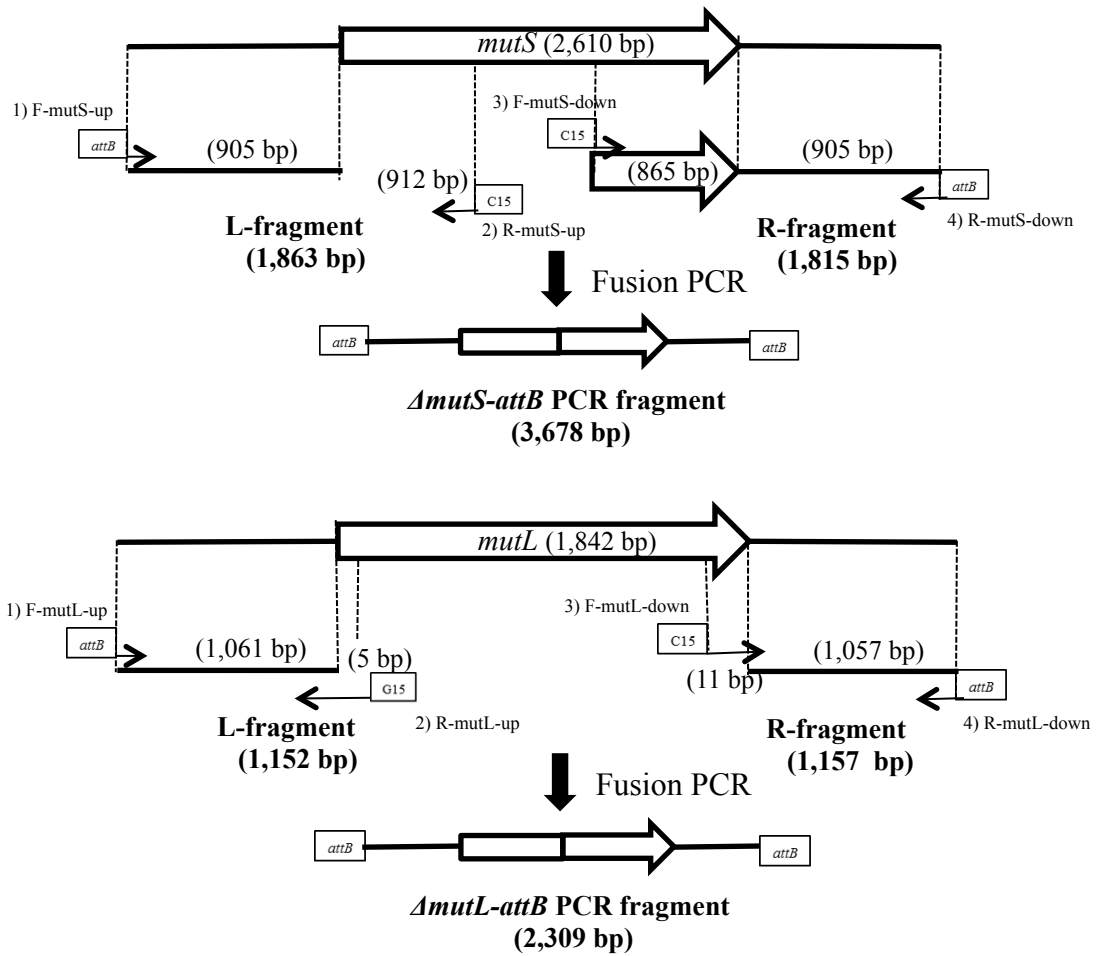

B

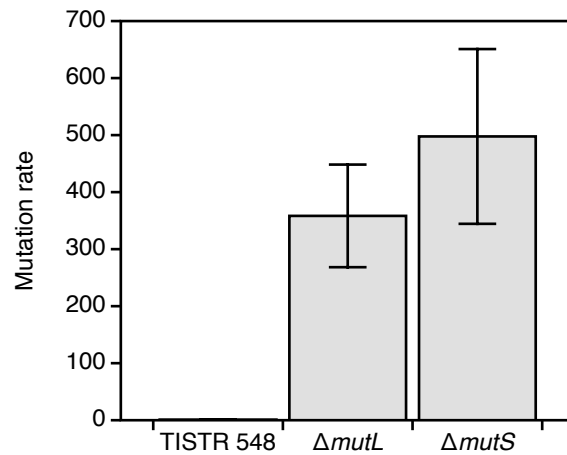

Supplement: S4 Fig — (A) An upstream DNA fragment including the coding sequence for the N-terminal portion of MutS and a downstream DNA fragment including the coding sequence for the C-terminal portion of MutS were separately amplified by PCR with the genomic DNA of TISTR 548 as a template and appropriate primers, and the two fragments were connected together by fusion PCR. The resultant ΔmutS-attB PCR fragment was inserted into pK18-attP, which was used for construction of ΔmutS and 200MΔmutS as described in the methods section. Similarly, ΔmutL and 200MΔmutL were constructed. (B) Mutation rate was determined as the ratio of apparent mutation frequency of ΔmutS or ΔmutL to that of their parent. (PDF) [file pone.0215614.s004.pdf]

**A**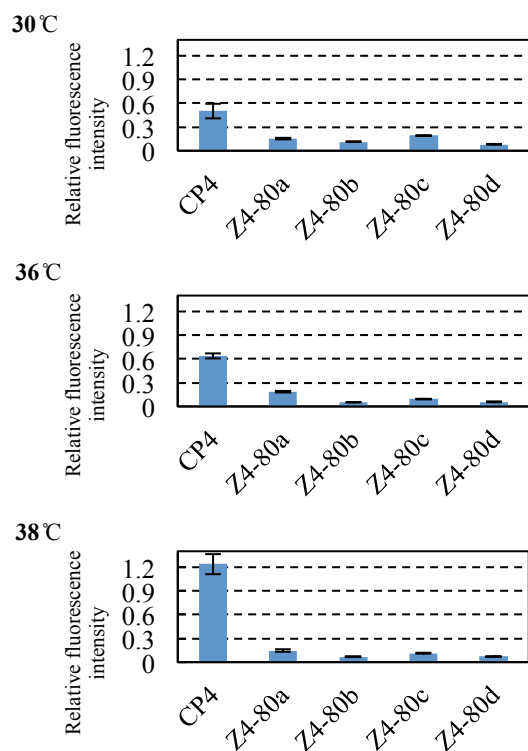**B**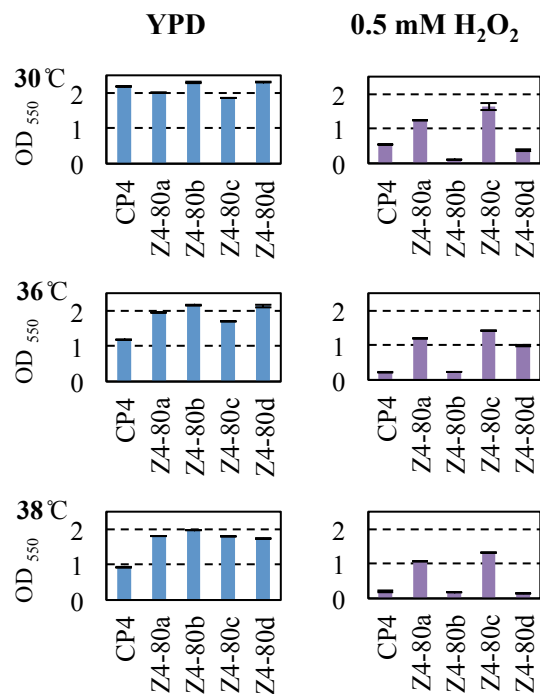**C**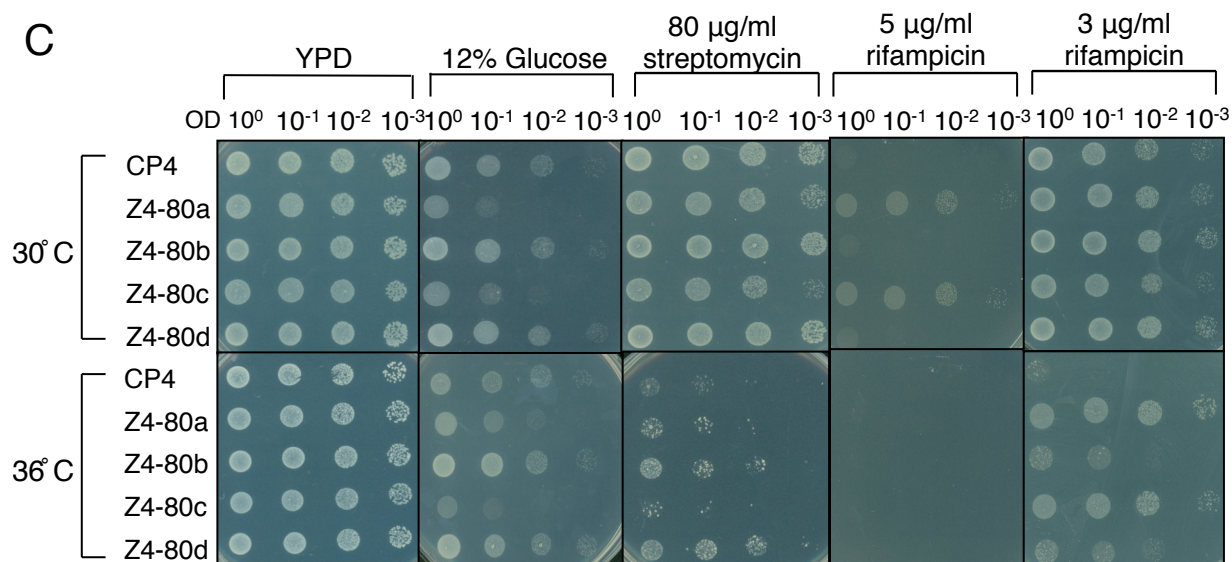**D**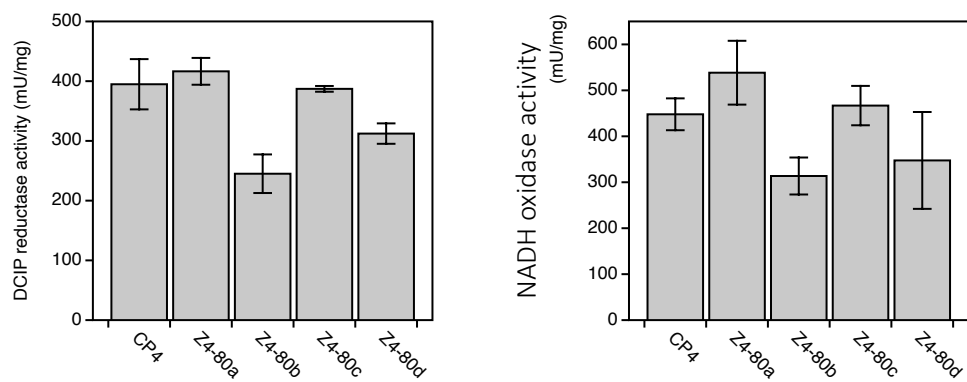

Supplement: S5 Fig — (A) Comparison in the accumulation of ROS. Thermoadapted mutants, Z4-80a, Z4-80b, Z4-80c and Z4-80d, from CP4 were grown at 30°C, 36°C and 38°C in YPD medium. Using cells at the exponential phase, fluorescent intensity with H2DCFDA, which reflects accumulation of ROS, was measured. (B) Resistance to H2O2 among thermoadapted mutants from CP4. Cells that were similarly grown at 30°C in TPD medium until the exponential phase were inoculated and grown at 30°C, 36°C and 38°C in YPD medium with or without 0.5 mM H2O2 for 24 h. The turbidity at 24 h was measured. (C) Effects of a high concentration of glucose and antibiotics on growth of thermoadapted mutants. Cells were grown at 30°C in YPD medium until the exponential phase. The culture was serially diluted and spotted on YPD plates containing 12% glucose or antibiotics and incubated at 30°C for 24 h. (D) Respiratory activities of thermoadapted mutants. Cells were grown at 30°C in YPD medium under a static condition, and their membrane fractions were prepared. NADH dehydrogenase (DCIP reductase) and NADH oxidase activities in the membrane fractions were then determined. (PDF) [file pone.0215614.s005.pdf]

A

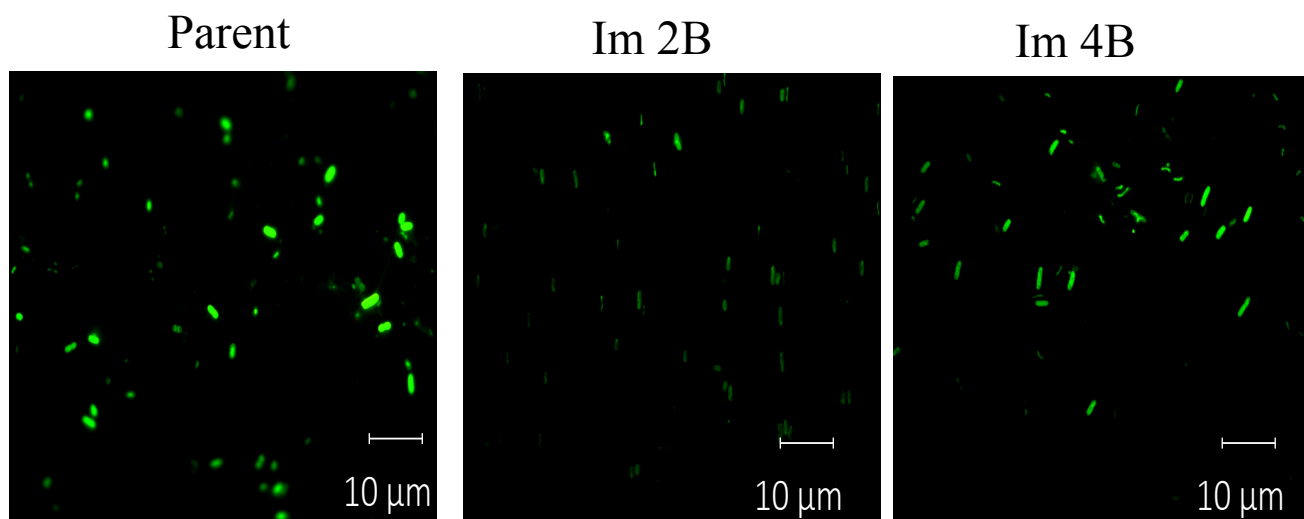

B

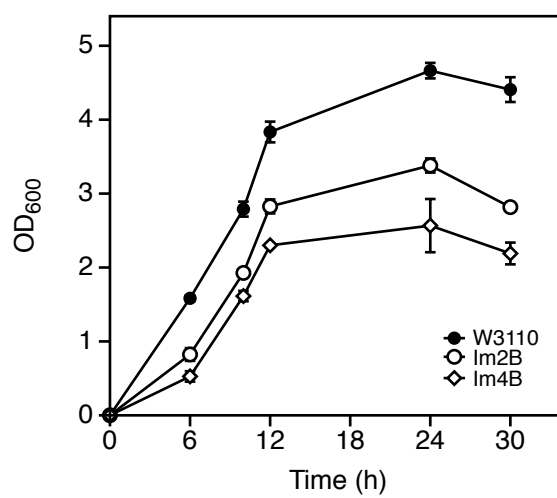

Supplement: S6 Fig — (A) Thermoadapted mutants, ImB2 and ImB4, from W3110 were grown at 47°C in LB medium and cells at the exponential phase were stained with acridine orange. (B) Cell growth was compared by cultivation at 37°C in LB medium. (PDF) [file pone.0215614.s006.pdf]
