# Supplementary material for: Capacity for survival in global warming: Adaptation of mesophiles to the temperature upper limit
Source: PLoS One. 2019 May 7;14(5):e0215614. doi: 10.1371/journal.pone.0215614 (PMC6504187; doi:10.1371/journal.pone.0215614)
Supplement: S1 Table — (PDF) [file pone.0215614.s007.pdf]

S1 Table. Summary of mutations in thermo-adapted mutants from *Zymomonas mobilis* TISTR548.

									Mutant	
Locus_tag	Gene	Classification	Product	Position	Reference	Alteration	Mutation	Amino acid change	200M	MAS1
-			-	97242-97307			Tn insertion		+	+
ZZ6_0121		Transcriptional regulation	DEAD/DEAH box helicase domain protein	130315	C	T	Transition	Gly110Arg		+
-			-	396944	C	G	Transversion		+	+
ZZ6_0448		Transporter	sodium/hydrogen exchanger	513196	G	A	Transition	Val242Ile	+	+
ZZ6_0464		Membrane stabilization	Phospho-N-acetylmuramoyl-pentapeptide- transferase	536297	C	T	Transition	Val179Met	+	+
ZZ6_0695		Transporter	Taurine-transporting ATPase	799465	C	T	Transition	Met417Ile	+	+
ZZ6_0717		Transcriptional regulation	signal transduction histidine kinase	827096-827129			Tn insertion	Split (Tn): aa14stop	+	+
ZZ6_0765		Membrane stabilization	GtrA family protein	868056-868086			Tn insertion	Split (Tn) aa18stop	+	+
ZZ6_0806		General metabolism	Formate--tetrahydrofolate ligase	921574	C	T	Transition	Ser492Phe	+	+
ZZ6_0951		Others	protein of unknown function DUF1239	1087369	C	CTAT	InDel	ATT insert as Ile in the next of Ala36	+	+
-			-	1091424	GCCCCCC	GCCCCC	InDel		+	+
-			-	1091426	C	G	Transversion		+	+
-			-	1091427	C	G	Transversion		+	+
-			-	1091643	GA	G	InDel		+	+
ZZ6_0986		Membrane stabilization	polysaccharide biosynthesis protein	1124869	C	T	Transition	Trp209Stop	+	+
-			-	1141264	T	A	Transversion		+	+
ZZ6_1055		General metabolism	pyruvate kinase	1210347	A	G	Transition	Met286Val		+
-			-	1235528	G	A	Transition		+	+
-			-	1285391-1285441			Tn insertion	Split (Tn)	+	+
ZZ6_1230		Protein quality control	thiol-disulfide isomerase	1405792	TG	T	InDel	Leu67fs: aa80stop	+	+
ZZ6_1423		Transcriptional regulation	TonB-dependent receptor	1611478-1611516			Tn insertion	Split (Tn)	+	+
ZZ6_1572		Transcriptional regulation	TonB-dependent receptor	1798985-1799049			Tn insertion	Split (Tn)	+	+
ZZ6_1657		Protein quality control	ATP-dependent chaperone ClpB	1893769	G	A	Transition	Pro774Leu		+
ZZ6_1692		Transcriptional regulation	two component transcriptional regulator, winged helix family	1935128	CCCCAAAG	C	InDel	Gly92fs: aa97stop	+	+
