# Supplementary material for: Capacity for survival in global warming: Adaptation of mesophiles to the temperature upper limit
Source: PLoS One. 2019 May 7;14(5):e0215614. doi: 10.1371/journal.pone.0215614 (PMC6504187; doi:10.1371/journal.pone.0215614)
Supplement: S2 Table — (PDF) [file pone.0215614.s008.pdf]

S2 Table. Summary of mutations in thermoadapted mutants from *Zymomonas mobilis* CP4.

									Mutant			
Locus_tag	Gene	Classification	Product	Position	Reference	Alteration	Mutation	Amino acid change	Z4-80a	Z4-80b	Z4-80c	Z4-80d
ZCP4_0028		Transcriptional regulation	signal transduction histidine kinase	32323	C	T	Transition	Met99Ile		+		
-			-	98768	G	GATGCCAGA	InDel					+
ZCP4_0125		Transporter	K+ transporter	142649	A	G	Transition	Thr403Ala	+			
ZCP4_0125		Transporter	K+ transporter	143231	C	T	Transition	Arg597Cys			+	
ZCP4_0567		Transcriptional regulation	DNA-directed RNA polymerase subunit beta	648575	G	A	Transition	Arg164Cys	+		+	
ZCP4_0588		General metabolism	formyltetrahydrofolate-dependent phosphoribosylglycinamide formyltransferase	670742	C	T	Transition	Leu118Phe				+
ZCP4_0707		Transporter	ABC-type nitrate/sulfonate/bicarbonate transport system, ATPase component	799915	G	A	Transition	Pro438Leu	+			
-			-	1205885	T	TCAGGTCGC	InDel				+	
-			-	1205931	T	TCGCAGGTCGCAGGT	InDel				+	
-			-	1714424	TTATT	TT	InDel				+	
-			-	1714426	A	AGGCT	InDel			+		
-			-	1714428	TC	TCTC	InDel			+	+	
ZCP4_1646		General metabolism	1-(5-phosphoribosyl)-5-((5-phosphoribosylamino)methylideneamino) imidazole-4-carboxamide isomerase	1856048	A	ATTTATGACGGCTCAC	InDel	IYDGSL insertion		+		
ZCP4_1702		Transporter	RND family efflux transporter, MFP subunit	1926213	G	A	Transition	Glu136Lys	+			
ZCP4_1702		Transporter	RND family efflux transporter, MFP subunit	1926238	C	T	Transition	Thr144Met			+	
ZCP4_1703		Transporter	efflux transporter, outer membrane factor lipoprotein, NodT family	1928285	A	G	Transition	Lys462Glu				+
ZCP4_1739		Membrane stabilization	sphingosine/diacylglycerol kinase-like enzyme	1966395	C	T	Transition	Ala89Val	+			
ZCP4_1739		Membrane stabilization	sphingosine/diacylglycerol kinase-like enzyme	1966601	C	T	Transition	Leu158Phe				+
ZCP4_1739		Membrane stabilization	sphingosine/diacylglycerol kinase-like enzyme	1966796	G	A	Transition	Val223Met			+	
