# Supplementary material for: Capacity for survival in global warming: Adaptation of mesophiles to the temperature upper limit
Source: PLoS One. 2019 May 7;14(5):e0215614. doi: 10.1371/journal.pone.0215614 (PMC6504187; doi:10.1371/journal.pone.0215614)
Supplement: S3 Table — (PDF) [file pone.0215614.s009.pdf]

S3 Table. Summary of mutations in thermoadapted mutants from *Escherichia coli* W3110.

									Mutant	
Locus_tag	Gene	Classification	Product	Position	Reference	Alteration	Mutation	Amino acid change	Im2B	Im4B
Y75_p0796	ybiW	General metabolism	pyruvate formate lyase	861430	A	G	Transition		+	
-	-		-	987574	G	T	Transversion			+
Y75_p0997	ycdT	Membrane stabilization	diguanylate cyclase	1093686	T	C	Transition	Val130Ala		+
-	-		-	1097560	TC	T	InDel			+
-	-		-	1097741	TC	T	InDel		+	
-	-		-	2866111	A	G	Transition			+
Y75_p2745	gcvA	Transcriptional regulation	DNA-binding transcriptional dual regulator	2940893	GA	G	InDel	Pro111fs		+
Y75_p3209	rpoC	Transcriptional regulation	RNA polymerase, beta prime subunit	3449152	T	G	Transversion	Asp727Ala	+	
Y75_p3236	pflC	General metabolism	pyruvate formate lyase II activase	3490291	T	TA	InDel	Lys45fs		+
Y75_p3311	ompL	Membrane stabilization	outer membrane porin L	3572737	T	C	Transition	Phe118Leu		+
Y75_p3331	trkH	Transporter	potassium transporter	3603496	A	T	Transversion	Val14Asp		+
Y75_p3524	spoT	Transcriptional regulation	bifunctional (p)ppGpp synthetase II and guanosine-3',5'-bis pyrophosphate 3'-pyrophosphohydrolase	3816251	C	T	Transition	Gly589Ser		+
Y75_p3524	spoT	Transcriptional regulation	bifunctional (p)ppGpp synthetase II and guanosine-3',5'-bis pyrophosphate 3'-pyrophosphohydrolase	3817018	A	G	Transition	Met333Thr	+	
-	-		-	4371270	GAA	G	InDel		+	
-	-		-	4375299	A	C	Transversion		+	
