# Supplementary material for: Capacity for survival in global warming: Adaptation of mesophiles to the temperature upper limit
Source: PLoS One. 2019 May 7;14(5):e0215614. doi: 10.1371/journal.pone.0215614 (PMC6504187; doi:10.1371/journal.pone.0215614)
Supplement: S4 Table — (PDF) [file pone.0215614.s010.pdf]

S4 Table. Primers used for construction of mutators.

Primer name	Sequence (5’->3’)	Purpose
F-mutS-up	GGGGACAAGTTTGTACAAAAAAGCAGGCTCGGCTGTGCTTGCTCGCTAATC	gene disruption
F-mutS-down	GGGGGGGGGGGGGGGAAGATCGGCTGTCAGAAGGC	gene disruption
R-mutS-up	CCCCCCCCCCCCCCCTCAAGGTCGTCATCCCGTTG	gene disruption
R-mutS-down	GGGGACCACTTTGTACAAGAAAGCTGGGTCAGTCACGACAGAAGGCGTTT	gene disruption
F-mutlL-up	GGGGACAAGTTTGTACAAAAAAGCAGGCTCGGTGGGGCTATTTCCGGCTTAAA	gene disruption
F-mutL-down	GGGGGGGGGGGGGGGCTTCTCCAGCTGCGATACGATT	gene disruption
R-mutL-up	CCCCCCCCCCCCCCCATGGGTAAAGCTCGGCCATAAA	gene disruption
R-mutL-down	GGGGACCACTTTGTACAAGAAAGCTGGGTCGGCCATCCCAAATTAGACCGTT	gene disruption
F-mutS2-up	GGGGACAAGTTTGTACAAAAAAGCAGGCTCGATGTAGCGGCTGTCCTGATG	gene disruption
F-mutS2-down	CCCCCCCCCCCCCCCTGAGCAAGTCAGCCAAGGAT	gene disruption
R-mutS2-up	GGGGGGGGGGGGGGGTGTGATCCTTCGTCGTCGTC	gene disruption
R-mutS2-down	GGGGACCACTTTGTACAAGAAAGCTGGGTCTAGGAAACAGCCTGCCAAGG	gene disruption
