# Supplementary material for: Capacity for survival in global warming: Adaptation of mesophiles to the temperature upper limit
Source: PLoS One. 2019 May 7;14(5):e0215614. doi: 10.1371/journal.pone.0215614 (PMC6504187; doi:10.1371/journal.pone.0215614)
Supplement: S5 Table — (PDF) [file pone.0215614.s011.pdf]

S5 Table. List of sequencing data and platform used in this study.

Strain	Reference strain	BioProject accession	DRA accession	BioSample accession	Sequence platform
CP4	*Zymomonas mobilis* subsp. mobilis CP4	PRJDB7156	DRA007031	SAMD00129443	Illumina MiSeq
Z4-80a	*Zymomonas mobilis* subsp. mobilis CP4	PRJDB7156	DRA007032	SAMD00129444	Illumina MiSeq
Z4-80b	*Zymomonas mobilis* subsp. mobilis CP4	PRJDB7156	DRA007033	SAMD00129445	Illumina Miseq
Z4-80c	*Zymomonas mobilis* subsp. mobilis CP4	PRJDB7156	DRA007034	SAMD00129446	Illumina Hiseq 2500
Z4-80d	*Zymomonas mobilis* subsp. mobilis CP4	PRJDB7156	DRA007035	SAMD00129447	Illumina Hiseq 2500
TISTR 548	*Zymomonas mobilis* subsp. mobilis TISTR 548	PRJDB7157	DRA007036	SAMD00129448	Illumina Hiseq 2500
200M	*Zymomonas mobilis* subsp. mobilis TISTR 548	PRJDB7157	DRA007037	SAMD00129449	Illumina Hiseq 2500
MAS1	*Zymomonas mobilis* subsp. mobilis TISTR 548	PRJDB7157	DRA007038	SAMD00129450	Illumina Hiseq 2500
W3110	*Escherichia coli* str. K-12 substr. W3110	PRJDB7158	DRA007039	SAMD00129451	Ion Torrent PGM
Im2B	*Escherichia coli* str. K-12 substr. W3110	PRJDB7158	DRA007040	SAMD00129452	Ion Torrent PGM
Im4B	*Escherichia coli* str. K-12 substr. W3110	PRJDB7158	DRA007041	SAMD00129453	Ion Torrent PGM
