# Supplementary material for: Capacity for survival in global warming: Adaptation of mesophiles to the temperature upper limit
Source: PLoS One. 2019 May 7;14(5):e0215614. doi: 10.1371/journal.pone.0215614 (PMC6504187; doi:10.1371/journal.pone.0215614)
Supplement: S6 Table — (PDF) [file pone.0215614.s012.pdf]

S6 Table Primers used for confirmation of mutation sites in coding regions of thermoadapted mutants from *Z. mobilis* TISTR548.

Primer_name	Sequence	Target_gene:Mutation_position
ZM548bs11105-Right	GCAGATTCCGCCAAGATG	ZZ6_0207:229843
ZM548bs11105-left	TGCAAGCCTACCTTTCCC	ZZ6_0207:229843
200M_335321-1F	GCCAACGTCAGGCCTTGA	ZZ6_0294:335321
200M_335321-1R	TCGAGAACCGGCGTATCG	ZZ6_0294:335321
ZM548bs10836-Right	ATGCTCTTCTCCATCGGC	ZZ6_0448:513196
ZM548bs10836-left	GTGGCCAATTTTCGCAAG	ZZ6_0448:513196
200M_536297-01F	CCGATAATCGCCCCCGTC	ZZ6_0464:536297
200M_536297-01R	TCGCGGTCTCTATCTCGCT	ZZ6_0464:536297
ZM548bs10576-Right	TCCGGTGACAATAGGCGT	ZZ6_0695:799465
ZM548bs10576-left	GCTGACCTCCCCCATCTT	ZZ6_0695:799465
ZM548bs10553-Right	CATGGAACCGGAATTTGC	ZZ6_0717:827096
ZM548bs10553-left	AGCGCTAAAGGGATTGCC	ZZ6_0717:827096
ZM548bs10503-Right	AAACCCAGCGACGATTCA	ZZ6_0765:868056
ZM548bs10503-left	AGGCGGAGATTGTCATCG	ZZ6_0765:868056
200M_898967-01F	ACCAGTGCGACAATCAGCT	ZZ6_0785:898945
200M_898967-01R	CGATGTTGGTTGAATCCGGT	ZZ6_0785:898945
200M_921574-01F	CAGGATCTTTGCGCCTCGA	ZZ6_0806:921574
200M_921574-01R	TATTTTCGGCGGCGGGAT	ZZ6_0806:921574
ZM548bsIG6-Right	ACCTTTAGACGACGGGGC	ZZ6_0850:964578
ZM548bsIG6-Left	TGGGCTTCTTGTTCTGCC	ZZ6_0850:964578
ZM548bs10288-Right	GCACCCGTCTATTGCACC	ZZ6_0951:1087369
ZM548bs10288-left	CATCCATAACGGCCTTGC	ZZ6_0951:1087369
ZM548bs10250-Right	ATTGGTTTTGGGGGCAAT	ZZ6_0986:1124869
ZM548bs10250-left	GGCGTATCTATGGTGCCG	ZZ6_0986:1124869
pyruvate sequence F	AGACGTCATCGAAGCCAAAG	ZZ6_1055:1210347
pyruvate sequence R	GGTTTCGGTGAAGACGATGA	ZZ6_1055:1210347
ZM548bs11855-Right	TTCCGGAACAGAATCCGA	ZZ6_1230:1405792
ZM548bs11855-left	ATTAGCTCCCTGTCGGGC	ZZ6_1230:1405792
200M_1611502-01F	ATGACCCCGGGGACTTCT	ZZ6_1423:1611478
200M_1611502-01R	TTCTTTACCCCAGCCGCG	ZZ6_1423:1611478
ZM548bs11481-Right	CATAATGGGTAACGCGGC	ZZ6_1572:1798985
ZM548bs11481-left	TGGAGGCATTGTCGAAGG	ZZ6_1572:1798985
ClpB sequence R	GTCTGACCATCACGAATAGTGC	ZZ6_1657:1893769
ClpB sequence F	GGGTCGGACAGTTGATTTTTCC	ZZ6_1657:1893769
ZM548bs11361-Right	TAAACCCACTCGCAACGG	ZZ6_1692:1935128
ZM548bs11361-left	CATGGAATGCTGGCTTTCTT	ZZ6_1692:1935128
