# Supplementary material for: Capacity for survival in global warming: Adaptation of mesophiles to the temperature upper limit
Source: PLoS One. 2019 May 7;14(5):e0215614. doi: 10.1371/journal.pone.0215614 (PMC6504187; doi:10.1371/journal.pone.0215614)
Supplement: S7 Table — (PDF) [file pone.0215614.s013.pdf]

S7 Table Primers used for confirmation of mutation sites in coding regions of thermoadapted mutants from *Z. mobilis* CP4.

Primer_name	Sequence	Target_gene:Mutation_position
ZCP4_0028_F	GCCATTTCATTCTGGCTACG	ZCP4_0028:32323
ZCP4_0028_R	ATACCGCCTATCAGGATGATG	ZCP4_0028:32323
ZCP4_0039_F	CGTTGCAGAATCTGGTCTTG	ZCP4_0039:44175
ZCP4_0039_R	CGAATGTCCATAAAGCGACG	ZCP4_0039:44175
ZCP4_0125_1_F	GTGTGCCGTTGATTATTCTCG	ZCP4_0125:142649
ZCP4_0125_R	GGGATAAGTGTCTGACGAGAG	ZCP4_0125:142649
ZCP4_0125_2_F	ATGGCTTCTATCGCCTGATC	ZCP4_0125:143231
ZCP4_0125_2R	CGGAGATTGGTCTCTAGTGG	ZCP4_0125:143231
ZCP4_0567_F	CAGCTTTCAGCGACATAAGG	ZCP4_0567:648575
ZCP4_0567_R	TGAGGACGGATATGTTTCAGG	ZCP4_0567:648575
ZCP4_0588_F	AGGATTATATGGCGACCAAGG	ZCP4_0588:670742
ZCP4_0588_R	GATTCTTTCACCTTCGCTCG	ZCP4_0588:670742
ZCP4_0707_F	ACATCGGTTTGGCTTGTTAC	ZCP4_0707:799915
ZCP4_0707_R	TTACCTCAGCCCAGAAATCG	ZCP4_0707:799915
ZCP4_1646_F	TTCTGTCACTGAGATGGCTG	ZCP4_1646:1856048
ZCP4_1646_R	CGGAATTAACGGCGACTTTG	ZCP4_1646:1856048
ZCP4_1702_1_F	GATGTTTCCGGTTTGGTCAC	ZCP4_1702:1926213
ZCP4_1702_1_R	CCCATGTAAAGGTTGGGTTG	ZCP4_1702:1926213
ZCP4_1703_F	AGCCGCTCTTACAACCTATAC	ZCP4_1703:1928109
ZCP4_1703_R	CATTCCTGCGTGATCTTGTTC	ZCP4_1703:1928109
ZCP4_1739_F	AAGAAGAACAGCGAGGGTAC	ZCP4_1739:1966395
ZCP4_1739_R	TGCGGATCTTGTTGAAACAC	ZCP4_1739:1966395
ZCP4_1739_F2	CGACAGCCATATCGTCAAAC	ZCP4_1739:1966601
ZCP4_1739_R	TCAGGCCATCAAGATCAGTC	ZCP4_1739:1966601
