# Supplementary material for: Capacity for survival in global warming: Adaptation of mesophiles to the temperature upper limit
Source: PLoS One. 2019 May 7;14(5):e0215614. doi: 10.1371/journal.pone.0215614 (PMC6504187; doi:10.1371/journal.pone.0215614)
Supplement: S8 Table — (PDF) [file pone.0215614.s014.pdf]

S8 Table Primers used for confirmation of mutation sites in coding regions of thermoadapted mutants from *E. coli* W3110.

Primer_name	Sequence	Target_gene:Mutation_position
ybiW_861430-F1	TCATCGTCGTTGCCGTAC	Y75_p0796:861430
ybiW_861430-R1	GGTGGCAAATGGGGCTAT	Y75_p0796:861430
ycdT_1093686-F1	AGGCGTTGAGGAAGTTGTT	Y75_p0997:1093686
ycdT_1093686-R1	ACCGAAAGCAAGGTTAACCA	Y75_p0997:1093686
ydgH-2seq-L	GGCGGTCACTCAATTAACGA	Y75_p2732:2925784
ydgH-2seq-R	CAGCGTATGCACGACAAACT	Y75_p2732:2925784
gcvA_2940893-F1	TTAACCCCAACTGTCGGG	Y75_p2745:2940893
gcvA_2940893-R1	CCAAGCCGCAGTAAGTCA	Y75_p2745:2940893
rpoC_2-L	TAACCGGAGTCATCAGATACCTTC	Y75_p3209:3449152
rpoC_2-R	TTTGCGGACCAGATCATGTACA	Y75_p3209:3449152
pflC_3490291-F1	GCCGCAGTCGCTGTAAAA	Y75_p3236:3490291
pflC_3490291-R1	AACAGCGCCCACAAGATT	Y75_p3236:3490291
ompL_3572737-F1	GGGCGCGGGTATTATGTT	Y75_p3311:3572737
ompL_3572737-R1	GTGGTACGGTTCGACGTT	Y75_p3311:3572737
trkH-4seq-L	AACCCCTCACGGGATTTAAGTT	Y75_p3331:3603496
trkH-4seq-R	ATCATCAGTTAACCGGCATTGA	Y75_p3331:3603496
spoT_4-L	CTGATGATTGAACATCTCCACCTTG	Y75_p3524:3816251
spoT_4-R	ACCTCAAGCGTGATGATTCTGT	Y75_p3524:3816251
spoT_2-L	ATCATCACGCTTGAGGTTTTTCAG	Y75_p3524:3817018
spoT_2-R	ACGGCTATCAGTCTTTGCACA	Y75_p3524:3817018
